# Testicular Size Discrepancy in a Man Evaluated for Vasectomy

**Published:** 2016-01-04

**Authors:** Nirmish Singla, Lakshmi Priya Kunju, Julie Marie Jorns

**Affiliations:** 1 Department of Urology, University of Texas Southwestern Medical Center, Dallas, TX 75390, USA; 2 Department of Pathology, University of Michigan, Ann Arbor, MI 48109, USA

A 49-year-old asymptomatic man presents for a vasectomy evaluation. His past medical history is noncontributory, and his family history is remarkable for prostate cancer in his father. On genitourinary examination, his left testicle is notably larger than his right testicle, with concern for a firm, nontender upper pole mass palpated on the left side. Scrotal ultrasound is performed and reveals numerous hypoechoic masses within the left testicle (Figure 1) and a normal right testicle. He undergoes left radical orchiectomy, revealing the histologic patterns shown (Figure 2).

There is no evidence of angiolymphatic invasion or extension of this pathology beyond the tunica albuginea. Pre-operative tumor markers are as follows: lactate dehydrogenase (LDH) 204 IU/L [normal range 120–240 IU/L], alpha-fetoprotein (AFP) 3.3 ng/mL [normal range <7.9 ng/mL], β-human chorionic gonadotropin (β-hCG) <2 mlU/mL [normal <5 mIU/mL]. Chest x-ray and CT scan of the abdomen and pelvis reveal no lymphadenopathy or evidence of metastases.

**WHAT IS THE DIAGNOSIS?**

A. Leydig cell tumor

B. Non-seminomatous germ cell tumor (NSGCT)

C. Seminoma

D. Testicular lymphoma

**DIAGNOSIS**

B. Non-seminomatous (mixed) germ cell tumor (NSGCT)

## DISCUSSION

Testicular neoplasms are uncommon, with 8,820 new cases estimated to arise nationwide in 2014 [1]. Most are germ cell tumors (GCT), half of which are pure seminomas and half non-seminomatous (NSGCT) [2]. Mixed GCTs containing both seminomatous and non-seminomatous components are classified as NSGCT. Patients with suspicious intratesticular lesions on ultrasonography should undergo radical orchiectomy, as the histopathology can provide prognostic information, while removal of the testis is usually curative [3].

**Figure 2:**
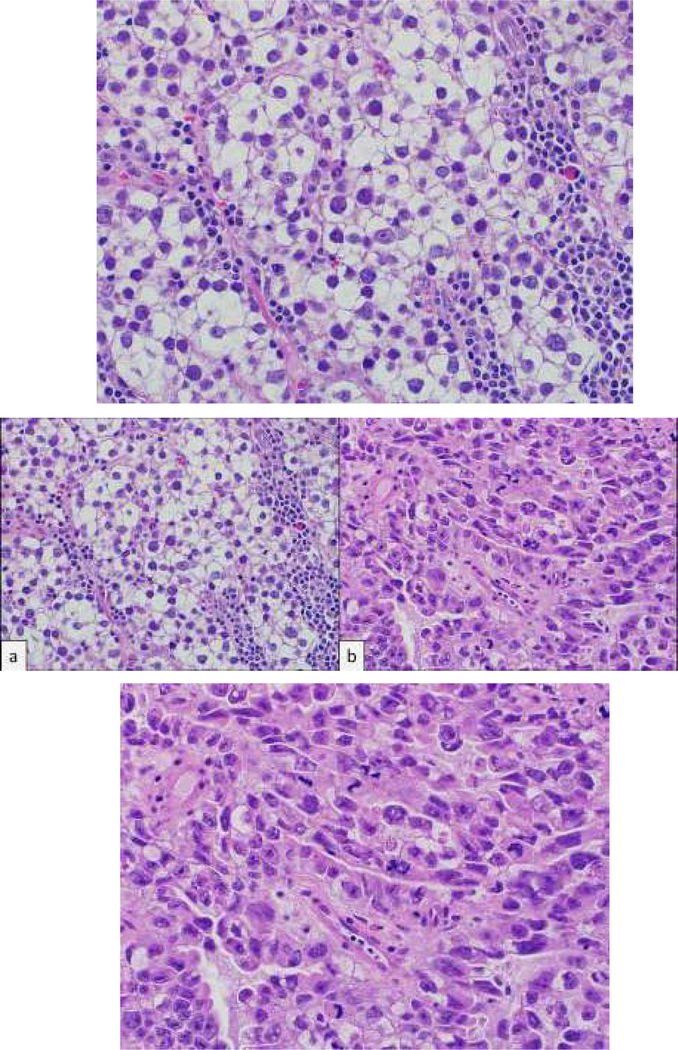
Histopathologic examination of left testis in two different areas (A, B) after radical orchiectomy. Histologic pattern reveals: A, Cells with clear cytoplasm, prominent nucleoli and lymphocytic fibrovascular septae. B, Primitive epithelial cells with crowded pleomorphic nuclei. Hematoxylin-eosin staining, original magnification x40.

This patient’s pathology revealed 80% seminoma (Figure 2(A)) and 20% embryonal carcinoma (EC, Figure 2(B)) components. Histologically, seminomas demonstrate nests of tumor cells with clear cytoplasm, prominent nucleoli and lymphocytic fibrovascular septae 4. EC appears less differentiated, with primitive epithelial cells and crowded pleomorphic nuclei [4]. EC is aggressive with high metastatic rates, yet tumor markers are often normal. The presence of an EC component or lymphovascular invasion poses greater risk for occult metastases.

The clinical stage of NSGCT holds prognostic value and directs management. The American Joint Committee on Cancer (AJCC) staging criteria rely on histopathology, tumor markers, and nodal or metastatic involvement on imaging.5 Tumor markers are useful in diagnosing GCTs and monitoring for inadequate treatment or recurrence; however, normal levels do not exclude GCTs. In this patient, the lack of angiolymphatic invasion or extension beyond the tunica albuginea classifies his pathologic stage as pT1 [5]. Taken together with no lymphadenopathy or metastases and normal tumor markers, his clinical stage is IA [5].

The optimal post-orchiectomy management for clinical stage I NSGCT remains controversial. While orchiectomy is curative in 70–80% of stage I NSGCT [3], retroperitoneal lymph node involvement has been reported in 25–35% of cases despite a normal CT [6]. The three acceptable treatment options—surveillance with routine tumor markers and CT reassessments, retroperitoneal lymph node dissection (RPLND), and chemotherapy—demonstrate equivalent long-term survival rates approaching 100%, and there are presently no published randomized trials directly comparing them [3,7-8]. Many centers employ a risk-adapted approach, in which patients at increased risk for relapse based on the presence of EC or lymphovascular invasion are encouraged to pursue RPLND or chemotherapy, whereas those without these features may prefer surveillance given their lower risk [7]. Of note, while radiotherapy is a standard treatment for seminomas, NSGCTs are radioresistant, and there is no role for adjuvant radiotherapy in their management [3].

The patient recovered uneventfully from his orchiectomy. He opted for active surveillance and demonstrated no evidence of relapse on CT imaging with normal serum tumor markers one year following his surgery.

## Figures and Tables

**Figure 1:**
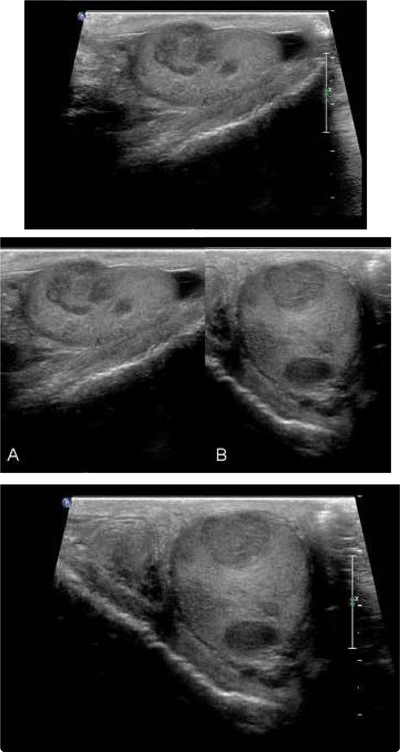
Scrotal ultrasound of left testis. Ultrasound reveals multiple hypoechoic masses within the left testicle, some abutting the tunica albuginea without frank extratesticular invasion. Shown in: A, longitudinal and B, transverse orientations.
